# The Rhizobia-*Lotus* Symbioses: Deeply Specific and Widely Diverse

**DOI:** 10.3389/fmicb.2018.02055

**Published:** 2018-09-12

**Authors:** María J. Lorite, María J. Estrella, Francisco J. Escaray, Analía Sannazzaro, Isabel M. Videira e Castro, Jorge Monza, Juan Sanjuán, Milagros León-Barrios

**Affiliations:** ^1^Estación Experimental del Zaidín, Consejo Superior de Investigaciones Científicas, Granada, Spain; ^2^Instituto Tecnológico de Chascomús, IIB-INTECH, UNSAM-CONICET, Chascomús, Argentina; ^3^Instituto Nacional de Investigação Agrária e Veterinária, Oeiras, Portugal; ^4^Facultad de Agronomia, Universidad de la República, Montevideo, Uruguay; ^5^Departamento de Bioquímica, Microbiología, Biología Celular y Genética, Universidad de la Laguna, Santa Cruz de Tenerife, Spain

**Keywords:** nitrogen fixation, bacterial diversity, inoculants, plant–microbe association, symbiosis

## Abstract

The symbiosis between *Lotus* and rhizobia has been long considered very specific and only two bacterial species were recognized as the microsymbionts of *Lotus*: *Mesorhizobium loti* was considered the typical rhizobia for the *L. corniculatus* complex, whereas *Bradyrhizobium* sp. (*Lotus*) was the symbiont for *L. uliginosus* and related species. As discussed in this review, this situation has dramatically changed during the last 15 years, with the characterization of nodule bacteria from worldwide geographical locations and from previously unexplored *Lotus* spp. Current data support that the *Lotus* rhizobia are dispersed amongst nearly 20 species in five genera (*Mesorhizobium, Bradyrhizobium, Rhizobium, Ensifer*, and *Aminobacter*). As a consequence, *M. loti* could be regarded an infrequent symbiont of *Lotus*, and several plant–bacteria compatibility groups can be envisaged. Despite the great progress achieved with the model *L. japonicus* in understanding the establishment and functionality of the symbiosis, the genetic and biochemical bases governing the stringent host-bacteria compatibility pairships within the genus *Lotus* await to be uncovered. Several *Lotus* spp. are grown for forage, and inoculation with rhizobia is a common practice in various countries. However, the great diversity of the *Lotus* rhizobia is likely squandered, as only few bacterial strains are used as inoculants for *Lotus* pastures in very different geographical locations, with a great variety of edaphic and climatic conditions. The agroecological potential of the genus *Lotus* can not be fully harnessed without acknowledging the great diversity of rhizobia-*Lotus* interactions, along with a better understanding of the specific plant and bacterial requirements for optimal symbiotic nitrogen fixation under increasingly constrained environmental conditions.

## Introduction

Legumes are remarkable for their ability to establish symbiosis with nitrogen-fixing bacteria known as rhizobia. Rhizobia infect the legume roots and trigger the formation of a new organ, the root nodule, specialized in N_2_ fixation. This highly energy-consuming process is supported by photosynthetically derived carbohydrate from the plant, and the rhizobia which in return provide the legume with ammonia. Thus, in association with the rhizobia legume plants are self-sufficient in nitrogen, which makes this symbiosis very important in ecological and economic terms.

*Lotus* is a cosmopolitan genus that includes more than 150 species (annual and perennial plants) with two major centers of diversity, the Mediterranean region (including portions of Europe, Africa, and Western Asia) and Western North America (Howieson et al., 2000; Sokoloff and Lock, 2005). Only a few *Lotus* species have been domesticated and improved by selection and plant breeding, to be used as forage for livestock, including *L. corniculatus* L*., L. uliginosus* Schkuhr. (also denoted *L. pedunculatus), L. tenuis* Waldst and Kit. (ex *L. glaber* Mill), *L. subbiflorus* Lagasca, and more recently *L. ornithopodioides* (Escaray et al., 2012). In addition, *L. japonicus* is used as a model for genetic and molecular studies (Márquez et al., 2005; Tabata and Stougaard, 2014).

A relatively small number of *Lotus* species have been studied regarding their root nodule symbionts, herein called the *Lotus* rhizobia. The symbiosis between *Lotus* and nitrogen-fixing rhizobia has been long considered highly specific and only two divergent groups of bacteria were traditionally recognized as the microsymbionts of the *Lotus* spp., the fast growers and the slow-growers. The “fast-growers” were classified as species *Mesorhizobium loti* (type strain NZP2213^T^) (Jarvis et al., 1982, 1997) and considered the typical rhizobial species for the *L. corniculatus* complex (i.e., *L. corniculatus, L. tenuis* and the model *L. japonicus*) (Jarvis et al., 1982; Saeki and Kouchi, 2000; Fulchieri et al., 2001). On another hand, the slow-growers named *Bradyrhizobium* sp. (*Lotus*) were typical symbionts for *L. uliginosus* (*pedunculatus*), *L. subbiflorus* and *L. angustissimus* and, until recently, have remained unclassified at species level.

This rather simple scheme likely derived from the fact that most *Lotus* rhizobia were initially isolated from agriculturally important *Lotus* species at few locations, far from centers of diversity (Jensen and Hansen, 1968; Monza et al., 1992; Irisarri et al., 1996; Fulchieri et al., 2001; Bullard et al., 2005). Those early studies of *Lotus* rhizobia focused on their agronomic potential as N_2_-fixing inoculants, whereas the recognition of bacterial diversity was not a priority (Monza et al., 1992; Irisarri et al., 1996; Fulchieri et al., 2001). This situation has dramatically changed during the last 15 years, as discussed below.

## Taxonomy and Diversity of the *Lotus* Rhizobia

### The *Mesorhizobium loti* Entanglement

**FIGURE 1:**
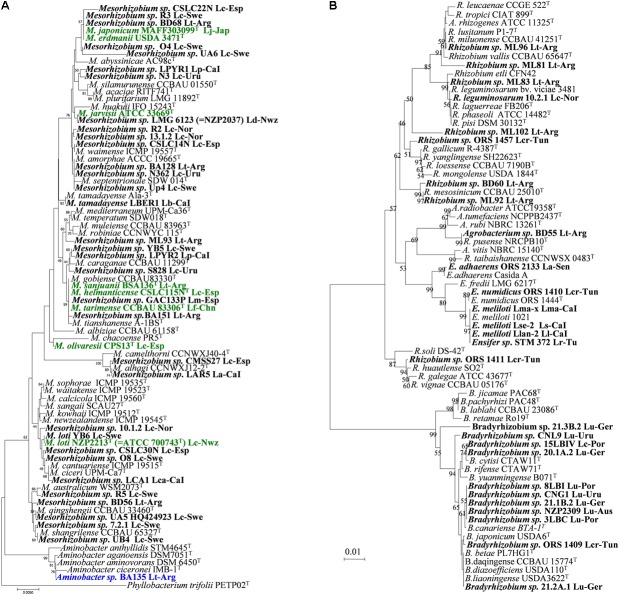
Taxonomy and phylogeny of rhizobial symbionts of *Lotus* spp. 16S rRNA phylogenetic tree including genera *Mesorhizobium* and *Aminobacter* **(A)**, and *Bradyrhizobia, Ensifer* (*Sinorhizobium*) and *Rhizobium* **(B)**. Bold names correspond to *Lotus* rhizobia, with original *Lotus* species and country of isolation indicated. In bold green are rhizobial species which have *Lotus* symbionts as type strains. Analyses were conducted with MEGA using Neighbor Joining Maximum Likelihood (NJML). A total of 1224 positions were used in the final dataset. La, *L. arinagensis*; Lar, *L. arabicus;* Lb, *L. berthelotii*; Lc, *L. corniculatus*; Lca, *L. campylocladus*; Lcr, *L. creticus*; Lf, *L. frondosus*; Lk, *L. kunkelii*; Lj, *L. japonicus*; Ll, *L. lancerottensis*; Lm, *L. maritimus*; Lma, *L. maculatus*; Lp, *L. pyranthus*. Lr, L. *roudairei*; Ls, *L. sessilifolius* ; Lt, *L. tenuis* (*L. glaber*)*;* Lu, *L. uliginosus* (*L. pedunculatus*). Arg, Argentina; Aus, Australia; Bel, Belgium; CaI, Canary Islands; Chn, China; Esp, Spain; Ger, Germany; Jap, Japan; Nor, Norway; Nwz, New Zealand; Por, Portugal; Sen, Senegal; Swe, Sweden; Tun, Tunisia; Uru, Uruguay. Accessions numbers for all sequences used in this review are listed in **Supplementary Table S1**.

Many so-called “*M. loti*” strains show significant differences in whole genomic DNA-DNA hybridization (Crow et al., 1981; de Lajudie et al., 1994) and in the 16S rRNA phylogeny (de Lajudie et al., 1998; Willems et al., 2001), indicating that they could belong to different species. The type strain NZP2213^T^ (deposited in other collections as ATCC 33669^T^= USDA 3471^T^ = IAM 13588^T^, LMG 6125^T^) was isolated from a root nodule of *L. corniculatus*, but great differences in 16S rRNA gene sequence have been reported for the bacteria deposited in different culture collections (Willems et al., 2001). These discrepancies have been explained by the presence of two different organisms in one of the subcultures, as well as by deficient long-term maintenance (Willems et al., 2001). The type strain ATCC 33669^T^ has been deaccessioned by the ATCC as type strain of *M. loti* and replaced by strain ATCC 700743^T^ (**Figure 1A**). Recently two novel species, *M. erdmanii* and *M. jarvisii*, have been proposed for the “*M. loti”* type strains USDA 3471^T^ and ATCC 33669^T^ (Martínez-Hidalgo et al., 2015), which are close relatives of the *M. opportunistum* and *M. huakuii* type strains and distant from *M. loti* NZP2213^T^ (see **Figure 1A**). In addition to the entanglement with the “type” strains, others so-called “*M. loti*” strains occupy distant phylogenetic branches, which suggests that they are also misclassified. Examples are strains MAFF303099 and R7A (ICMP 3153), two of the best studied *Lotus* symbionts. MAFF303099 was re-classified as *M. huakuii* (Turner et al., 2002; Wang et al., 2014). However, a more recent taxonomic characterization has placed strains MAFF303099 and R7A (ICMP3153) in the novel species *M. japonicum* (Martínez-Hidalgo et al., 2016). Similarly, other reference “*M. loti*” strains, like NZP2037 ( = LMG 6124), also show a large phylogenetic distance from the type strain *M. loti* NZP2213^T^ (**Figure 1A**), indicating the need of better characterizations and perhaps re-classification.

### Diversity of Rhizobia From Cultivated *Lotus* spp.

Several studies of rhizobia nodulating *L. corniculatus* from different locations and habitats in Europe and in South America have shown that most of the isolates falled within genus *Mesorhizobium*, but an unexpectedly great diversity of species, different to *M. loti*, was observed.

*Lotus corniculatus, L. maritimus* and *L. tenuis* nodule isolates from saline soils of Granada (Southeast Spain) were related to different species of the genus *Mesorhizobium*, such as *M. tarimense/M. tianshanense, M. chacoense/M. albiziae* and *M. alhagi* (Lorite et al., 2010b; see **Figure 1A**). Some of these rhizobia have been recently included in the new species *M. olivaresii* (Lorite et al., 2016). No *M. loti*-like bacteria were found in that study. A great genetic diversity has also been observed among strains nodulating *L. corniculatus* from Northwest Spain. These were distributed in five divergent phylogenetic groups within the genus *Mesorhizobium*, two of them related to *M. jarvisii* and *M. erdmanii* but the rest were phylogenetically divergent to the currently described *Mesorhizobium* species, and none was related with the type strain *M. loti* NZP2213^T^ (Marcos-García et al., 2015). Some of these isolates have been classified in the new species *M. helmanticense* (Marcos-García et al., 2017). On the contrary, most of the *L. corniculatus* nodule isolates from Norway and Sweden soils were initially grouped with *M. loti* NZP2213^T^, whereas only a minority of isolates were related to other species: *M. huakuii, M. tianshanense, M. plurifarium, M. amorphae* and *M. septentrionale* (Ampomah and Huss-Danell, 2011; Gossmann et al., 2012). However, this phylogenetic analysis did not include several *Mesorhizobium* species. We have observed that should *M. qingshengii, M. ciceri/M. canturiense, M. australicum* and *M. shangrilense* have been included in the 16S rRNA phylogenetic tree, most of the Scandinavian isolates would cluster closer to these later species than to *M. loti* NZP2213^T^ (see **Figure 1A**). This indicates that further studies are needed to assess the phylogenetic relationships of the Scandinavian strains. Many isolates from *L. corniculatus* wild plants in Belgium and Norway (De Meyer et al., 2011; Gossmann et al., 2012) grouped with *Mesorhizobium*, whereas others were close to various species in the *Rhizobium* genus. However host specificity of the Belgian strains was not tested, and Norwegian isolates failed to induce nodules in their original host, with the exception of *R. leguminosarum* 10.2.1 (*Rl* Norway), which induced nodule organogenesis in certain *Lotus* species (Gossmann et al., 2012). Diverse non-nodulating bacteria have been frequently isolated from nodules of *L. corniculatus* [e.g., *Burkholderia* spp. (Gossmann et al., 2012) or the recently described species *Phyllobacterium loti* S658^T^ (Sánchez et al., 2014)]. None of these bacteria were able to nodulate, although many could be recovered from nodules if co-inoculated along a truly nodulating partner, indicating their hitchhiker nature. Other non-rhizobium Gram-positive bacteria were isolated from *L. corniculatus* in Sweden such as *Rhodococcus* spp. and *Geobacillus* spp., and a symbiotic phenotype in their original host and *nodC* gene amplification was obtained (Ampomah and Huss-Danell, 2011). Nevertheless, these results should be confirmed. As mentioned above, several reports have shown the presence of non-nodulating bacteria inside the nodules (Gossmann et al., 2012; Sánchez et al., 2014), and the possibility exists, even following the appropriate methodology, to carry over the non-nodulating bacteria mixed with nodulating ones, especially when the companion has a duplication time significantly shorter than the true rhizobia (Sánchez et al., 2014).

A great majority of the isolates from *L. corniculatus* in Uruguay (Sotelo et al., 2011) were described as close relatives of species *M. huakuii*, and the rest were closely related to either *M. septentrionale* or *M. caraganae* (**Figure 1A**). Again, no *M. loti*-like strains were isolated in that study. Among 103 root nodule isolates from *L. tenuis* trap plants grown in soils of the Salado River Basin (Argentina), a majority were related to genus *Mesorhizobium*: only 2 isolates seemed closely related to *M. loti* NZP2213^T^, and the rest were more related to *M. amorphae, M. mediterraneum, M. tianshanense* or to strain NZP2037 (Estrella et al., 2009; see **Figure 1A**). Some of those have been classified in the new species *M. sanjuanii* (Sannazzaro et al., 2018). A large portion of the isolates were grouped within genus *Rhizobium*, close to typical common bean symbionts *R. gallicum, R. etli*, and *R. tropici* (**Figure 1B**). Also in that study (Estrella et al., 2009), a member of genus *Aminobacter* with symbiotic capacity was reported for the first time (BA135, **Figure 1A**).

Species like *L. uliginosus, L. subbiflorus* or *L. angustissimus* have been known to be nodulated by slow-growing bradyrhizobia (Cooper et al., 1985; Irisarri et al., 1996). Evidence suggests that the symbionts of *Lotus heermannii, L. micranthus*, and *L. strigosus* are also bradyrhizobia (Sachs et al., 2009). But only a few recent reports have studied genetic diversity and phylogenetic relationships of isolates from nodules of *L. uliginosus*, collected from cultivated fields in countries like Uruguay (Batista et al., 2013), or from wild plants in various European countries, such as Portugal (Lorite et al., 2012), Belgium (De Meyer et al., 2011) and Germany (Gossmann et al., 2012). These studies supported affiliation of these bacteria to genus *Bradyrhizobium*, in some cases to species *B. japonicum* (**Figure 1B**). The data indicate a more reduced biodiversity and wider geographical distribution than the *L. corniculatus* rhizobia. Exceptionally, strain Lulit12 related phyllogeneticaly to the broad host range *Mesorhizobium* *sp*. NZP2037 was isolated from *L. pedunculatus* in Uruguay (Batista et al., 2013). Also a non-bradyrhizobial strain belonging to the genus *Curtobacterium*, has been isolated from *L. pedunculatus* in Germany but reinoculation on the original host was not done (Gossmann et al., 2012).

### Diversity of Rhizobia From Non-cultivated *Lotus* spp.

The characterization of symbionts from wild *Lotus* species in different sites/countries has revealed additional diversity. The root nodule bacteria from a Mexican population of *Lotus oroboides* showed a great diversity of mesorhizobial strains that were related to *M. loti, M. amorphae, M. plurifarium* and *M. huakuii* (Qian and Parker, 2002). The nodule bacteria from *Lotus arabicus, L. argenteus, L. creticus, L. pusillus* and *L. roudairei* in Tunisian soils spread over four genera, *Mesorhizobium, Bradyrhizobium, Ensifer* ( = *Sinorhizobium*) and *Rhizobium* (Zakhia et al., 2004; Rejili et al., 2009, 2012). The bradyrhizobial strains from *L. creticus* were close to *B. yuanmingense* (Zakhia et al., 2004). Furthermore, some sinorhizobia from *L. creticus* (**Figure 1B**) were affiliated to the novel species *Ensifer numidicus* (Merabet et al., 2010).

Two novel mesorhizobial species, *M.* *tarimense* and *M. gobiense* were described amongst the root nodule rhizobia of *L. frondosus* and *L. tenui*s in soils of Xinjiang, China (**Figure 1A**). These bacteria are able to nodulate also *L. corniculatus* as well as other non-*Lotus* species like *Glycyrrhiza uralensis, Oxytropis glabra*, and *Robinia pseudoacacia* (Han et al., 2008a). No *M. loti* strains were isolated either from this region of China. Although *R. multihospitium* is associated with a quite broad range of legume species within ten genera, including the *Lotus* species *L. tenuis* and *L. frondosus*, nodulation of these hosts could not verified after reinoculation (Han et al., 2008b). On the other hand, the rhizobia from *Lotus* species endemic to the Canary Islands (*L. lancerottensis, L. kunkelii, L. arinagensis, L. pyranthus, L. berthelotii, L. calli-viridis* and *L. campylocladus*) were affiliated with *Mesorhizobium* (**Figure 1A**), *Ensifer* (**Figure 1B**) or *Rhizobium/Agrobacterium* (**Figure 1B**) (Lorite et al., 2010a). The mesorhizobial strains were close to *M. plurifarium, M. caraganae, M. ciceri, M. alhagi* and the novel species *M. tamadayense*, isolated from *L. berthelotii* and *Anagyris latifolia* (Ramírez-Bahena et al., 2012). Neither *M. loti* nor *Bradyrhizobium* were identified among the Canarian *Lotus* isolates. Moreover, *Ensifer* *meliloti*, the typical species nodulating alfalfa, was described for the first time as a *Lotus* symbiont, representing the new symbiovar lancerottense (León-Barrios et al., 2009) (**Figure 1B**). The new species *Phyllobacterium* *salinisoli* was also isolated from a root nodule of *L. lancerottensis*, however, reinfection was unsuccessful and PCR amplification of *nodC* gene failed (León-Barrios et al., 2018).

In summary, the characterization of symbionts from worldwide geographical locations and from unexplored *Lotus* spp. have revealed an extraordinary diversity amongst the *Lotus* symbionts, overwhelmingly wider than the simple taxonomic scheme *M. loti/Bradyrhizobium* sp. which has been acknowledged for many years and is still assumed in many laboratories. Current data support that the *Lotus* rhizobia are dispersed among five genera: *Mesorhizobium, Bradyrhizobium, Rhizobium, Ensifer*, and *Aminobacter*. *Bradyrhizobium* spp. are the characteristic symbionts for species like *L. uliginosus, L. subbiflorus, L. angustissimus* and likely a few other *Lotus* spp. Some of these *Lotus* bradyrhizobia are related to *B. japonicum* or *B. yuanmingense*, although detailed taxonomic characterization await in most cases. The importance of genus *Rhizobium* as true symbionts of *Lotus* remains to be clarified since re-infections or presence of nodulation genes have either not been confirmed or failed in most cases (Zakhia et al., 2004; Han et al., 2008b; Estrella et al., 2009; Lorite et al., 2010a). Hitherto only few strains from Argentina and one isolate of *R. leguminosarum* (Estrella et al., 2009; Gossmann et al., 2012) have been proved as true *Lotus* symbionts. Genus *Ensifer* has been frequently isolated from wild *Lotus*, and it could be the predominant microsymbiont in arid regions (Zakhia et al., 2004; León-Barrios et al., 2009; Rejili et al., 2009, 2012; Lorite et al., 2010a; Merabet et al., 2010).

Rhizobia from the *L. corniculatus*/*L. japonicus* complex usually belong to *Mesorhizobium* genus. In addition to the initial *M. loti* (Saeki and Kouchi, 2000; Saeki and Ronson, 2014), new mesorhizobial species have been defined to accomodate *Lotus* rhizobia, such as *M. jarvisii, M. erdmanii, M. japonicum, M. olivaresii* and *M.* *helmanticense* (from *L. corniculatus*), *M. tarimense* and *M. gobiense* (from *L. frondosus* and *L. tenuis*), *M. sanjuanii* (from *L. tenuis*) and *M. tamadayense* (from *L. berthelotii*). Furthermore, several *Lotus* rhizobia representing distinct lineages await detailed characterization and new species will likely be described in the future. *Lotus* symbionts belonging or related to other previously described mesorhizobial species have been also reported: *M. huakuii, M. amorphae, M. mediterraneum, M. tianshanense, M. alhagi, M. ciceri, M. plurifarium* and *M. caraganae*. Also important, *M. loti* appears an infrequent *Lotus* symbiont, as it seems absent from many diverse soils and locations. As a consequence *M. loti* should no longer be considered as the preferred *Lotus* symbiont.

## Specificity of the Rhizobia-*Lotus* Symbioses

### The *Lotus*-Rhizobia Symbiotic Compatibility Groups

Measured with the old and simplistic rhizobial taxonomic scheme, two *Lotus* symbiotic compatibility groups have been traditionally described: the group I included species such as *L. corniculatus, L. tenuis* or *L. japonicus* which could establish effective nitrogen-fixing symbiosis with the so-called “*M. loti”* and other mesorhizobial species; and *Lotus* group II, including *L. uliginosus, L. subbiflorus*, and *L. angustissimus*, effectively nodulated by bradyrhizobia. Efficient bacteria in one compatibility group were usually non-nodulating, non-infective, inefficient or poorly efficient in the other group, with the exception of a few broad-host-range strains like *Mesorhizobium* *sp*. NZP2037, which could form moderately effective nitrogen-fixing nodules with both group I and II *Lotus* species (Pankhurst et al., 1987; Monza et al., 1992; Saeki and Kouchi, 2000; Lorite et al., 2010a). Supporting this scheme is the fact that no bradyrhizobia have been isolated from *L. corniculatus, L. tenuis* or other group I species (Han et al., 2008a; Estrella et al., 2009; Lorite et al., 2010a,b; De Meyer et al., 2011; Gossmann et al., 2012; Batista et al., 2013).

There has also been significant confusion in the description of the symbiotic phenotypes resulting from certain plant-bacteria combinations. For instance, Pankhurst (1970) reported that *L. corniculatus* mature nodules, induced either by strain *Bradyrhizobium* sp. NZP2309, *M. loti* NZP2213^T^ or the broad host range *Mesorhizobium* sp. NZP2037, were all externally similar and contained infected cells, despite *Bradyrhizobium* sp. NZP2309 being inefficient or partially efficient in *L. corniculatus* (Lorite et al., 2012; Gossmann et al., 2012). In a more recent report, *L. japonicus* inoculated with *Bradyrhizobium* sp. NZP2309 produced delayed and poorly efficient nitrogen-fixing nodules, which had few intact symbiosomes and senesced early (Bek et al., 2010). In contrast, strains of genus *Mesorhizobium* (NZP2213^T^, R7A, etc.) inoculated on *L. uliginosus* (and also on *L. angustissimus* and *L. subbiflorus*) formed supernumerary small and uninfected root structures that in some occasions were called nodules (and thus were assigned a Nod^+^Fix^-^ phenotype; Irisarri et al., 1996; Díaz et al., 2005), whereas other authors called such formations outgrowths, pseudonodules, or tumor-like structures (and therefore were qualified as Nod^-^; Pankhurst, 1970; Bek et al., 2010). Before going further we should make a clear distinction between such structures, and propose to name pseudonodules to structures (tumor-like, bumps, outgrowths, etc.) which are induced but not invaded by the bacterium and therefore do not contain bacteria or bacteroids inside. In contrast, a nodule should always contain invading bacteria, which may or may not differentiate into nitrogen-fixing bacteroids, and therefore may be Fix^+^ or Fix^-^. Pseudonodules should be also distinguished from the so-called spontaneous nodules that may appear in some legumes in the absence of rhizobia (Truchet et al., 1989).

The increasing number of *Lotus* species studied and the description of additional bacterial species and genera as natural symbionts of *Lotus* spp. has opened a wider and more complex scenario than the classical two *Lotus* symbiotic compatibility groups. For instance, a likely third compatibility group can be envisaged which would comprise *Lotus* species endemic to the Canary Islands, like *L. lancerottensis, L. sessilifolius*, and *L. maculatus.* This group is efficiently nodulated by fast-growing strains of *E. meliloti* sv. lancerottense which are unable to fix nitrogen with either of the above *Lotus* groups I or II (León-Barrios et al., 2009; Lorite et al., 2010b). Mesorhizobia isolates from *L. arinagensis* and *L. kunkelii* in the Canary Islands seem incompatible with both *L. corniculatus* and *L. uliginosus*, but also with *L. lancerottensis* (Lorite et al., 2010b) and perhaps represent another incompatibility group. Also mesorhizobia strains isolated from *L. oroboides* in Mexico failed to nodulate *L. corniculatus* although unfortunately no other *Lotus* were tested (Qian and Parker, 2002). Neither of the Australian commercial inoculant strains for *L. uliginosus* (CC829) and *L. corniculatus* (SU343) can fix nitrogen with the promising forage species *L. ornithopodioides* (Howieson et al., 2011). Indeed, these authors found no strains compatible with all the 15 *Lotus* species evaluated. Attention should be paid to the possibility that some of the above mentioned bacteria-host incompatibilities be actually due to genotype-specific, rather than species-specific, host–rhizobia interactions. For instance, varying levels of nitrogen-fixing efficiency in their original hosts have been observed amongst rhizobia from *L. subbiflorus* and *L. uliginosus* in Uruguay and Portugal, respectively (Irisarri et al., 1996; Lorite et al., 2012). Similar results were observed amongst *L. corniculatus* rhizobia from Spain and Norway, ranging from highly efficient to non-nodulating, depending on the plant cultivar used (Lorite et al., 2010b; Gossmann et al., 2012). Thus, we recommend that more than one plant genotype be tested before claiming that a given bacterium is not compatible with a particular *Lotus* host.

The existence of incompatibility groups within the genus *Lotus*, together with other reasons like the consideration by several authors of *L. corniculatus* as an archetype *Lotus* species, have created additional confusion about the relationships of *Lotus* rhizobia with the traditional cross-inoculation groups. Isolates from *L. uliginosus* were shown to fix nitrogen with *Ornithopus sativus* but were inefficient on *Astragalus glycyphyllus* (Jensen, 1967). Rhizobia from *Lupinus luteus* were found to induce efficient nodules on *L. uliginosus* (Jensen and Hansen, 1968). In contrast, the commercial strain NZP2309 and other *L. uliginosus* isolates from Portugal formed inefficient nodules on *L. luteus* (Lorite et al., 2012). In addition, NZP2309 and several Uruguayan bradyrhizobia isolated from *L. uliginosus* nodules were efficient or partially efficient with *Spartium junceun* (Batista et al., 2013). As detailed below, the Portuguese and Uruguayan bradyrhizobia were shown to carry symbiotic genes closely related to the symbiovar genistearum, where *Spartium, Genista, Ornithopus*, and *Lupinus* rhizobia are included (Vinuesa et al., 2005; Lorite et al., 2012; Batista et al., 2013).

On the other hand, *L. corniculatus* rhizobia fixed nitrogen efficiently with *Anthyllis vulneraria*, but inefficiently with *A. glycyphyllus* and *O. sativus* (Jensen, 1967). In addition, certain rhizobia from *Lupinus densiflorus* were effective in *L. corniculatus* and *A. vulneraria* and ineffective in *O.* *sativus* and *L. uliginosus* (Jensen, 1967), while *A. vulneraria* and *A. glycyphyllus* rhizobia were efficient on *L. corniculatus* but inefficient on *L. uliginosus* and *O. sativus* (Jensen and Hansen, 1968). Furthermore the *M. loti* NZP2213^T^ was unable to nodulate *Leucaena leucocephala, Carmichaelia flageliformis, O. sativus*, and *Clianthus puniceus* (Pankhurst et al., 1987).

Besides the *bona fide* symbionts which have been originally isolated from a *Lotus* spp., a number of other rhizobia have been shown to form nodules (sometimes nitrogen-fixing) with certain *Lotus* spp. The *P. vulgaris* symbiont *R. etli* CE3 is able to induce nodulation in *L. japonicus*, although these nodules could not sustain nitrogen fixation and senesced early (Cárdenas et al., 1995; Banba et al., 2001). Several broad-host range rhizobia such as *Ensifer* *fredii* NGR234, *R. tropici* CIAT 899 and *R. leucaenae* CFN299 are able of inducing low or moderately efficient nodules in *L. japoniucs* and/or *L. corniculatus* (Hernández-Lucas et al., 1995; Schumpp et al., 2009). In contrast to NGR234, another *S. fredii* strain HH103 can form nitrogen-fixing nodules on *L. burtii* but forms only small uninfected outgrows in *L. japonicus* Gifu (Sandal et al., 2012). Broad host-range strains have been also isolated from *Lotus*, i.e., *Mesorhizobium* sp. NZP2037 which is able to fix nitrogen on its natural host *L. divaricatus* as well as *L. japonicus, L. corniculatus* and *L. uliginosus*, but also on *L. leucocephala, C. flageliformis, O. sativus* and *Clianthus puniceus* (Pankhurst et al., 1987; Scott et al., 1987).

### Horizontal Transfer of Symbiotic Genes

Genetic variability plays an important role in the evolution of symbiotic interactions and the horizontal gene transfer (HGT) represents a powerful mechanism for the continuous evolution of host-microorganism relationships. Genes that transfer horizontally can be found in a variety of mobile genetic elements (MGE) such as plasmids, transposons, bacteriophages, integrons, insertion sequences and genomic islands. Through some of the aforementioned elements, HGT is responsible for the great diversity of rhizobial symbionts, currently distributed in more than one hundred species in some 14 genera of two bacterial classes, α- and β-proteobacteria (Peix et al., 2015). In all cases examined symbiotic genes have been found in the so-called accessory genome, associated with MGE such as the large plasmids preferentially found in genera like *Rhizobium* or *Ensifer*, or chromosomal symbiosis islands common in mesorhizobia and bradyrhizobia.

Horizontal gene transfer can be inferred from phylogenies of symbiotic genes, but has also been observed in the field. Sullivan et al. (1995), after introducing *L. corniculatus* inoculated with the *M. japonicum* R7A in a New Zealand grassland with no history of *Lotus* cultivation, detected the spread of the inoculant’s symbiosis genes into several indigenous bacterial species which were thus converted into symbionts. These authors were the first to demonstrate that non-symbiotic mesorhizobia can evolve into symbionts in a single step, by acquisition of a 500-kb MGE that was called “symbiosis island” in analogy to the pathogenicity islands of bacterial pathogens (Sullivan and Ronson, 1998). In addition to strain R7A (502 kb; Ronson et al., 2002), two other symbiosis islands have been sequenced from *Lotus* mesorhizobia: a 611-kb symbiosis island was revealed in the chromosome of strain MAFF303099 (Kaneko et al., 2000), and more recently the symbiosis island of the broad host range strain NZP2037, isolated from *L. divaricatus* (533 kb; Kasai-Maita et al., 2013). Interestingly, the core regions of the three MGEs involved only 165 of the more than 500 genes carried by each island (Kasai-Maita et al., 2013). One of the more striking differences is the presence of a type-four secretion system (T4SS) in R7A and NZP2037 instead of the T3SS of MAFF303099; nevertheless it seems that both T3SS and T4SS are involved in nodulation efficiency and symbiotic host range, and could be therefore considered functionally analogous (see below).

**FIGURE 2:**
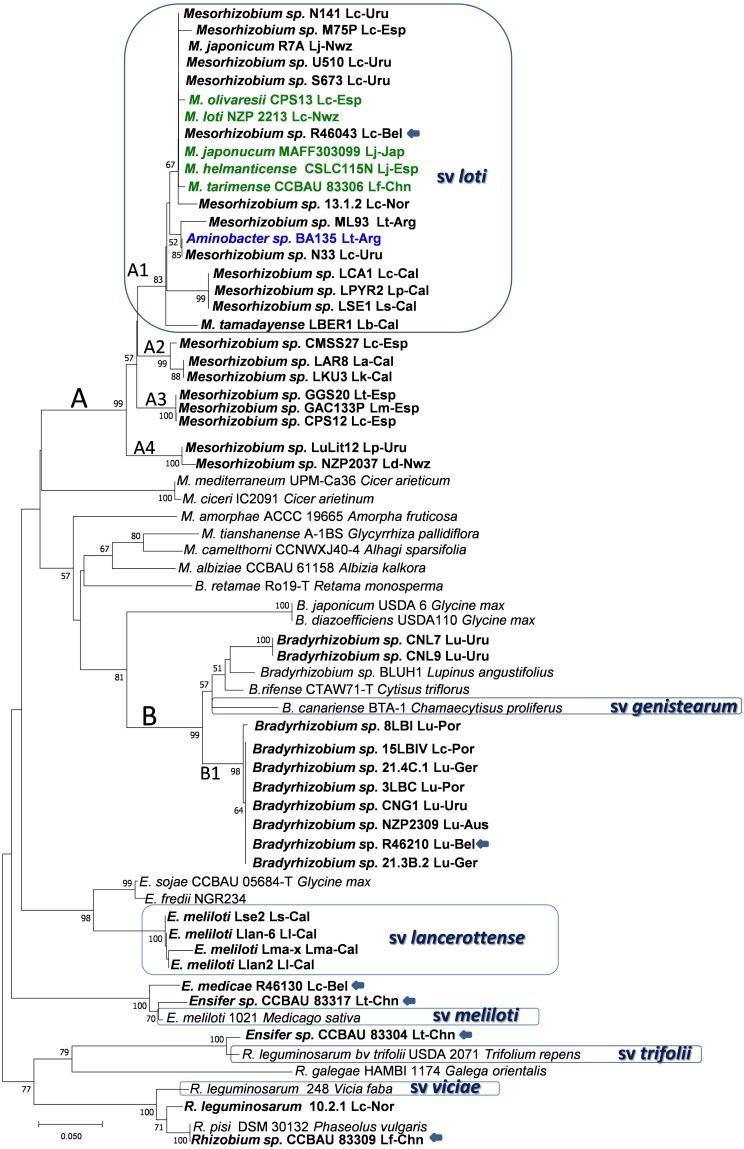
Phylogenetic tree of *nodC* gene sequences. Bold names correspond to *Lotus* rhizobia, with original *Lotus* species and country of isolation indicated. In bold green are rhizobial species which have *Lotus* symbionts as type strains. Previously defined symbiovars are indicated. Strains with unconfirmed symbiotic phenotypes in *Lotus* are indicated with an arrow. Analyses were conducted with MEGA using Neighbor Joining Maximum Likelihood (NJML), for a total of 203 positions. Abbreviations and accesions numbers are as in **Figure 1**.

Besides the pioneer studies of C. Ronson’s Laboratory (Ramsay and Ronson, 2015), phylogenetic evidence suggest that the symbiosis island is frequently exchanged among *Mesorhizobium* species, as symbiotic genes are highly conserved in different species of this genus (see below **Figure 2**). Bradyrhizobial symbionts of the *L. pedunculatus* complex species are also presumed to carry symbiosis islands, although this extreme has not been confirmed yet. Since different species carry highly conserved *nod* and *nif* genes (Lorite et al., 2012; Batista et al., 2013) it can be suspected that HGT is playing also a role in the preferential spread of the symbiosis genes within the genus *Bradyrhizobium*.

### Symbiovars

Strains MAFF303099 and R7A induce efficient nitrogen-fixing nodules in *L. japonicus* and *L. corniculatus* and their symbiosis islands share conserved backbone sequences of 248 kb, with 98% of nucleotide sequence identity (Sullivan et al., 2002). Rhizobia harboring symbiotic sequences that cluster with MAFF303099 and R7A are referred to as symbiovar loti (Turner et al., 2002; Rogel et al., 2011). To this symbiovar belong most of the mesorhizobia isolated from *L. corniculatus* and related species (e.g., *L. tenuis, L. japonicus, L. frondosus, L. maritimus, L. oroboides*), including the type strain *M. loti* NZP2213^T^, which shares above 98% of symbiotic sequences with MAFF303099 and R7A (**Figure 2** and **Supplementary Figure S1**). The data suggest that symbiovar loti is widely extended among the symbionts of *L. corniculatus, L. japonicus* and related species, supporting that the loti symbiotic island is frequently exchanged among *Mesorhizobium* species. It is unclear whether transfer and establishment of the symbiosis island is more efficient among species of this genus or whether mesorhizobia are preferred symbionts for the *L. corniculatus* group. Nevertheless, bacteria from other genera may carry the symbiovar loti symbiotic genes, as it was reported for a true nodulating *Aminobacter* isolate (Estrella et al., 2009), indicating that transfer is a frequent event that enable diverse bacteria with symbiotic traits and other adaptive functions to become efficient symbionts for this group of *Lotus* spp.

On the other hand, *Mesorhizobium* sp. NZP2037, another *L. corniculatus* and *L. japonicus* symbiont which has a *nodC* sequence with < 93% similarity to those of the symbiovar loti clade, occupied a distinctive cluster (branch A4, **Figure 2**). This strain also shows a broader host range, so it likely represents a symbiovar different from loti. This may also be the case for other branches (i.e., branch A2, **Figure 2**) that include strains unable to nodulate or which formed poorly efficient nodules in *L. corniculatus*, such as strains LKU3 and LAR8, two isolates from *L. kunkeli* and *L. arinagensis* (Lorite et al., 2010a), strain CMSS27 from *L. corniculatus* (Lorite et al., 2010b), as well as the one formed by GGS20, CPS12 and GAC133P, isolated from *L. tenuis, L. corniculatus* and *L*. *maritimus* respectively (branch A3, **Figure 2**). Additional studies are necessary to establish the host range of these bacteria and whether they could represent distinctive symbiovars.

The *Lotus* bradyrhizobial symbiotic genes seem to have a quite different phylogenetic history. Less variability is found among the slow-growing rhizobia, although *nodC* (**Figure 2**) and *nifH* (**Supplementary Figure S1**) sequences spread in either of two clusters closely related to the symbiovar genistearum (Gossmann et al., 2012; Lorite et al., 2012; Batista et al., 2013). The larger cluster (B1) could be a new symbiovar specific of the *Lotus* bradyrhizobia (**Figure 2**).

A different picture is provided by several *Lotus* rhizobial populations isolated from saline soils in Africa and the nearby Canary Islands. The nodulation genes of a group of *Ensifer meliloti* isolated from various *Lotus* spp. in the Canaries had the closest *nodC* phylogenetic relatives in *S. fredii* NGR234 and USDA 257. This together with a distinctive *nifH* genotype and specific host range defined the symbiovar lancerottense (León-Barrios et al., 2009), appreciably divergent from symbiovars loti or genistearum (**Figure 2**). The *nod* gene sequences of *Ensifer* isolates from *L. arabicus* in Senegal, and from *L. creticus* and *L. roundairei* in Tunisia were placed within different symbiovars among the *Ensifer* lineages, but distantly from other *Lotus* isolates. It is worth noting that the *Lotus* sinorhizobia have all been isolated from arid areas where drought, high temperatures and salinity are frequent abiotic stresses. Also related to symbiovars meliloti, trifolii and viciae seem some *Lotus* isolates from China (Han et al., 2010) (**Figure 2**), as well as strain R-46130 isolated from *L. corniculatus* which had a *nodC* gene sequence close to symbiovar meliloti albeit no symbiotic phenotype was verified (De Meyer et al., 2011). Care must be paid before assigning unverified symbiotic phenotypes to bacteria isolated from nodules, even if they seem to carry nodulation genes. Despite carrying *nod* genes related to bv. viciae and producing Nod factors recognized by *L. japonicus* at the molecular level, several *R. leguminosarum* isolates from *L. corniculatus* were unable to induce nodulation in most *Lotus* spp. and only formed non-fixing nodules in *L. burttii* (Gossmann et al., 2012), suggesting that these bacteria are not compatible symbionts of any *Lotus* spp.

### Bacterial Compounds Involved in Symbiotic Specificity

#### Nod Factors

Effective nodule formation results from the expression of the host legume-bacteria compatibility through a complex process involving the exchange of specific signals between plant and bacteria (Dénarié et al., 1996; Radutoiu et al., 2003). The recognition between both symbionts initiates with the perception of specific plant flavonoids that attract the rhizobia to the root and activate expression of the nodulation genes. The specificity of this initial stage is mediated by the protein NodD, a LysR-type transcriptional activator of genes (*nod, nol, noe)* necessary for synthesis and export of the Nodulation Factors (NF) (Caetano-Anollés and Gresshoff, 1991; Dénarié et al., 1996; Spaink, 2000). Chemical structure of the NF consists of an oligomeric backbone of β-(1,4) linked *N*-acetyl-D-glucosaminyl (GlucNAc) residues, with a fatty acyl group attached to the nitrogen of the non-reducing saccharide (Spaink, 2000). Given the similarity of the oligosaccharide backbone to chitin fragments, NF are often called lipo-chitin oligosaccharides (LCOs) (Spaink, 2000). Rhizobia appear to produce complex mixtures of LCOs, which differ in the number of GlucNAc residues, the nature of the fatty acyl group as well as the substituents at the non-reducing- and/or reducing-terminal residues.

Nodulation factors mediate nodule induction by all rhizobia except some NF-independent *Bradyrhizobium* strains that initiate the symbiosis by an alternative mechanism (Giraud et al., 2007). Infection via intracellular root hair infection threads (IT) is the common way of infection in *Lotus* spp. However, a crack entry based mode of entry that involves Nod factor perception in the root cortex has also been described in some *Lotus* spp. (James and Sprent, 1999; Madsen et al., 2010; Acosta-Jurado et al., 2016). Moreover, a NF-independent and IT-independent single cell infection mechanism has also been reported in certain *L. japonicus* mutants (Madsen et al., 2010).

The symbiosis between rhizobia and the *Lotus* has been traditionally considered highly specific, as discussed above. The chemical structures and biological activities of the NFs produced by several mesorhizobial *Lotus* symbionts have been described (López-Lara et al., 1995; Olsthoorn et al., 1998; Niwa et al., 2001), as well as those by a bradyrhizobial strain (NZP2039; Bek et al., 2010). It must be mentioned that the identities of the *Lotus* *nod*-inducing compounds are yet unknown. The recent discovery of a differential activation of *nod* genes by the transcriptional regulators NodD1 and NodD2 suggest that plant-derived inducing compounds can vary upon specific infection stages (Kelly et al., 2018). So far, NF produced by *Lotus* rhizobia have been identified in NodD-modified strains that recognize heterologous flavonoids. Various mesorhizobial strains, all symbionts of *L. corniculatus*/*L. japonicus*, produce different mixtures of LCOs where the common component is a GlucNAc pentasaccharide in which the non-reducing terminal residue is modified with a C18:1 (vaccenic) fatty acid, *N*-methylated and carbamoylated, whereas the reducing end is substituted with 4-*O*-acetyl-fucose (López-Lara et al., 1995; Olsthoorn et al., 1998; Niwa et al., 2001; Bek et al., 2010). In contrast, the LCOs produced by strain NZP2037 bear an additional carbamoyl group on the non-reducing terminal GlucNAc residue (López-Lara et al., 1995). Lack of carbamoylation of the NF had no effect in nodulation (Rodpothong et al., 2009). In contrast, R7A *nodZ* and *nodL* mutants, producing NF without the acetylfucose on the reducing end displayed a host-specific phenotype, forming uninfected nodule primordia in *L. filicaulis* and *L. corniculatus*, but effective nodules in *L. japonicus* (Rodpothong et al., 2009). More recently, Bek et al. (2010), also making use of a naringenin-inducible NodD activator system used by other authors, identified the structure of Nod Factors produced by *Bradyrhizobium* sp. NZP2309, an effective symbiont of *L. uliginosus*, and also determined more precisely the position of the decorations of the NF produced by strain R7A. The analysis revealed closely related but not identical structures for the major types of LCOs produced by the mesorhizobial strain R7A and the *Bradyrhizobium* sp. NZP2309. The main difference was the presence of two carbamoyl groups in the non-reducing GlucNAc of the NZP2039 NF, instead of only one in the mesorhizobial compounds. NZP2309 NF would be similar to those of the broad host range strain NZP2037, further supporting that carbamoylation at the non-reducing end of NF is not related with host range. Indeed, both NF types seem to be similarly recognized by either *L. japonicus* or *L. uliginosus*, indicating that NF perception is not responsible for the known symbiotic incompatibilities. Thus, additional components are likely necessary for infection of nodule primordia and progression of symbiotic establishment (Bek et al., 2010).

#### Exopolysaccharides

Legumes inoculated with compatible rhizobia form, in response to rhizobial Nod factors, nodule primordia in the root cortex which are targeted for bacterial infection. Infection of most legumes is initiated when bacteria become entrapped by a curled root hair. Local cell wall hydrolysis and invagination of the plant plasma membrane lead to formation of tubular structures, the IT. ITs are colonized by dividing bacteria and proceed through the epidermal cell layer into the root cortex before ramifying in the underlying nodule primordia. Rhizobia are released from ITs into plant cells and enclosed in membrane-delimited compartments called symbiosomes.

Bacterial cell surface components and particularly exopolysaccharides (EPSs) play important roles during root nodule infection, although their precise functions have not been yet resolved. EPSs have been proposed to play active roles as signals to mitigate plant defense responses, but also to protect bacteria against harshness imposed by changing osmotic conditions or against host-derived reactive oxygen species (Niehaus et al., 1993; Parniske et al., 1994; Wielbo et al., 2004). Rhizobial EPS are species- or strain-specific, homo- or heteropolysaccharides which are secreted into the external environment, or retained at the bacterial surface as capsular polysaccharides (CPS). A large diversity of EPS chemical structures can be found amongst rhizobia, regarding sugar composition, linkages, repeating unit size and degree of polymerization, as well as non-carbohydrate substitutions (Downie, 2010). The *exo* genes responsible for the production, polymerization, and export of EPS in most rhizobial species are clustered in plasmids, although in *Lotus* symbionts these genes are chromosomal (Kaneko et al., 2000).

The importance of EPS for the infection process has been well established in the *S. meliloti*-alfalfa and *R. leguminosarum*-pea symbioses, where indeterminate-type nodules are formed. EPS-defective bacterial mutants are impaired in IT formation and elongation, resulting in the formation of uninfected pseudonodules (Pellock et al., 2000; Stuurman et al., 2000; Laus et al., 2004, 2005). The addition of low amounts of purified wild-type EPS often complement and rescue the infective capacity in EPS-deficient mutants, suggesting that EPS are signaling molecules rather than providing a structural role during IT development (Djordjevic et al., 1987; Battisti et al., 1992; Urzainqui and Walker, 1992).

Unlike the vast amount of information concerning EPS function in indeterminate nodules, the role of EPS in legumes that form determinate nodules, such as *Lotus* spp, has been elusive. The first studies carried out with EPS-deficient mutants of the broad host range strain NZP2037, suggested that EPS is not required for infection of determinate nodules because these mutants still formed effective nodules on the compatible host *L. pedunculatus* but failed to elicit nodules with the indeterminate host *Leucaena leucocephala* (Hotter and Scott, 1991). Likewise, Tn5 mutagenesis of MAF303099 revealed that 32 mutants in EPS synthesis have a symbiotic phenotype indistinguishable of the wild-type on *L. japonicus* (Mishima et al., 2008). The dispensability of EPS to establish symbiosis with *L. japonicus* resembled other rhizobial species such as *R. etli* (Borthakur et al., 1986; Diebold and Noel, 1989), *S. fredii* (Kim et al., 1989) and *B. japonicum* (Law et al., 1982), infecting legumes with determinate nodules. These reports again supported the idea that EPS are essential to infect indeterminate hosts but not for determinate nodule hosts. However, some questions arose from the delayed nodulation phenotype or the severe symbiotic defects shown by some EPS mutants of *B. japonicum* on soybean (Parniske et al., 1993; Becker et al., 1998). More recently, Kelly et al. (2013) have characterized EPS from R7A and isolated Tn*5* mutants affected in various steps of EPS biosynthesis, evaluating their symbiotic phenotypes on two *Lotus* species. Chemical analysis has revealed that R7A produces an *O*-acetylated acidic EPS which has an octosaccharide repeating unit composed of glucose, galactose, glucuronic acid and riburonic acid residues (Kelly et al., 2013; Muszyński et al., 2016). Mutants affected in early biosynthetic steps (*exoA* and *exoB*) formed nitrogen-fixing nodules on *L. corniculatus* and *L. japonicus* ‘Gifu,’ whereas mutants affected in mid or late biosynthetic steps (*exoO, exoU, exoK, mlr5265*, and *mlr5266*) were impaired on both hosts, with the severity of the symbiotic defect depending on the particular mutant. The *exoU, exoO*, and *mlr5265* mutants exhibited the most severe symbiotic defects and induced the formation of small, uninfected nodule primordia. These mutants were disrupted at the stage of IT development (Kelly et al., 2013). This phenotype resembled that of EPS mutants of *S. meliloti* and *R. leguminosarum* which are impaired in their symbiosis with indeterminate nodule-forming hosts. Kelly et al. (2013) suggested that full-length wild-type EPS acts as a positive signal modulating plant defense responses and allowing IT development. In contrast, truncated EPS would be perceived by the host as a negative signal inducing defense reactions and blocking IT development. Strains like *exoB* mutants which produce no EPS, would somehow avoid plant surveillance and form infected nodules, albeit with lower efficiency than the wild-type strain (Kelly et al., 2013). More recently, J. Stougaard and co-workers have identified the *L. japonicus* EPS receptor EPR3, involved in monitoring the EPS status of rhizobia during infection (Kawaharada et al., 2015). EPR3 can bind monomeric EPS and would be able to distinguish between EPS variants to act positively (toward sustained infection) in response to compatible EPS, or negatively in the presence of incompatible EPS. The finding that induction of *Epr3* gene expression is dependent on Nod Factors and that EPR3 does not affect NF-mediated signaling, provides strong support to the hypothesis that rhizobial access to legume roots is controlled by a two-step mechanism (Kawaharada et al., 2015). Future studies shall reveal whether different *Lotus* spp. possess distinct *Epr3* alleles and if recognition of EPS by EPR3 is involved in *Lotus*-rhizobia compatibility. The characterization of EPS produced by bradyrhizobial strains like NZP2309 should certainly help to this aim.

#### Secreted Proteins

Many gram negative bacteria use the type III (T3SS) or type IV (T4SS) protein secretion systems to invade host cells. Experimental evidence from different microorganisms showed that both systems are involved in protein translocation and modulation of the host defenses (Chisholm et al., 2006; Desveaux et al., 2007).

Several rhizobia such as *B. japonicum* USDA110, *S.* *fredii* NGR234 and other *S. fredii* strains, use T3SS to secrete effectors and infect host cells. Among *Lotus* symbionts, it was observed that strain MAF303099 possesses a T3SS whereas strain R7A contains a T4SS (Sullivan et al., 2002; Hubber et al., 2004). Interestingly, MAFF303099 seems to have had a T4SS in the past but lost it by deletion and gained a T3SS at a different location within its symbotic island (Sullivan et al., 2002). Hubber et al. (2004) showed that a T4SS seems the most common feature amongst *Lotus* mesorhizobia, albeit T3SS and T4SS would be functionally exchangeable. The expression of rhizobial T3SS components and secreted effectors (Nops) are usually induced by flavonoids via the TtsI transcriptional activator, whose expression is regulated by NodD (Krause et al., 2002; Marie et al., 2004). Studies by Sánchez et al. (2012) revealed that MAFF303099 has a functional T3SS which is probably regulated by flavonoids. Hubber et al. (2007) also evidenced that R7A *vir* genes are subject to a two-tiered regulatory cascade in response to symbiotic signals, likely plant flavonoids, first through NodD via the *nod* box upstream of *virA* and then through VirA/VirG two-component regulatory system. Genetic synteny suggests a similar regulation of T4SS in the broad host range strain NZP2037 (Kasai-Maita et al., 2013).

Several candidate T3SS effector proteins have been identified in rhizobia: NopD, NopL, NopM, NopP, and NopT (Bartsev et al., 2004; Skorpil et al., 2005; Rodrigues et al., 2007; Dai et al., 2008; Kambara et al., 2009). However, genes potentially encoding these proteins are not present in MAFF303099, except for Mlr6316 which has 50% similarity to NopD (Rodrigues et al., 2007), although other T3SS effector genes have been identified flanking the MAFF303099 T3SS cluster (Sánchez et al., 2012).

Only two proteins, Msi059 and Msi061, have been shown to be transported through the T4SS of *M. loti* R7A (Hubber et al., 2004). Interestingly, Msi059 displays putative functional conservation with NopD, a predicted C48 cysteine peptidase (Rodrigues et al., 2007). On its hand, Msi061 shows some homology to *A. tumefaciens* VirF protein which participates during T-DNA translocation into the plant cells (Schrammeijer et al., 2001).

The effect of T4SS and T3SS mutations of *Mesorhizobium* strains on the symbiosis with *Lotus* species and other legumes has been analyzed by different authors. The symbiotic capacity of a NZP2037 mutant lacking 11 *vir* genes was essentially comparable to that of the wild-type strain, suggesting a limited contribution of T4SS to the host range among *Lotus* species (Kasai-Maita et al., 2013). In contrast, in R7A mutations in *vir* genes or in *msi059* gene have host-dependent effects, negatively affecting nodule formation on usual legume hosts as *L. corniculatus*, but allowing active nodule formation in legume species which normally are not nodulated by the wild-type, such as *Leucaena leucocephala* (Hubber et al., 2004). These host-dependent effects are similar to those reported for T3SS mutants of MAFF303099 (Hubber et al., 2004). The host-dependence of the symbiotic phenotypes may reflect differences in the host reactions to secreted effector proteins (Hubber et al., 2004), or to the dual effect of certain effectors (Sánchez et al., 2012). Thus, in *L. leucocephala* the effector proteins may induce plant defense responses, analogous to the avirulence proteins of plant pathogens, resulting in abortion of infections; whereas in *L. corniculatus* they may facilitate infection, perhaps by suppressing plant defense reactions (Marie et al., 2001; Hubber et al., 2004), or by interfering with the process of autoregulation of nodulation (Nelson and Sadowsky, 2015) with the aim of increasing nodulation.

### Plant Compounds Involved in Symbiotic Specificity

#### Plant Genetic Control of the Symbiosis

Upon recognition of specific rhizobial Nod factors, a host genetic program is initiated which governs the symbiosis with rhizobia. This program coordinates two parallel developmental processes in legume roots: *de novo* cortical cell division leading to nodule primordia formation, and the IT initiation in the root hairs guiding bacteria toward dividing cortical cells (reviewed in Binder et al., 2014).

Quite a significant number of proteins involved in recognition and binding of NF (NFR1 and NFR5), signal transduction from the plasma membrane to the nucleus (i.e., SYMRK), including nuclear and perinuclear calcium spiking and decoding the calcium signal, as well as several transcription factors involved in early symbiotic responses, have been identified in *L. japonicus* and other legumes (reviewed in Binder et al., 2014; Liang et al., 2014). Many of such proteins also play a key role in the recognition and response to arburcular mycorrizal fungi, and therefore are components of a genetic program that is common for rhizobia and AM fungi symbioses.

Bacterial infection via IT is mediated by the bacterial EPS and the *L. japonicus* EPR3 LysM receptor kinase. EPR3 is able to discriminate between wild-type and truncated or abnormal bacterial EPS (Kawaharada et al., 2015). Expression of EPR3 is dependent on previous Nod factor perception and signal transduction, and is tissue specific which suggests that EPR3 perception of compatible EPS promotes an intracellular cortical infection mechanisms to maintain bacteria enclosed in plant membranes (Kawaharada et al., 2017). As mentioned above, it would be interesting to know if EPR3 is involved in discriminating EPS of compatible and incompatible rhizobia in *L. japonicus* and other *Lotus* spp.

Also, host plant can control nodule number and new infection throught genetic mechanisms. Three *L. japonicus* *CLAVATA3/ESR*-related genes are involved in limiting the nodule number per root system; meanwhile a *NRSYM1* transcriptional factor has been described as regulator of nitrate-induced mechanisms during nodulation (Nishida et al., 2018).

#### Condensed Tannins

Condensed tannins (CTs) are polymeric end-products of the flavonoid biosynthetic pathway synthesized through the condensation of flavans, a diverse group of metabolites based on a heterocyclic ring system derived from the phenylalanine amino acid (reviewed by Ferreira and Slade, 2002; Marles et al., 2003). These compounds are the most abundant secondary metabolites in plants and are known as proanthocyanidins, as they yield anthocyanidins when depolymerized under oxidative conditions (Porter et al., 1985). CTs are present in fruits, bark, leaves and seeds of many plants and are of major importance in many agricultural crops. For instance, the flavor and astringency of wine are principally determined by both the levels and composition of CTs present in the grape skin (Gawel, 1998). CTs play different roles in plants, the best known are the protection against microbial pathogens, insects and larger herbivores (reviewed by Dixon et al., 2005). In most plant species, CTs are accumulated in the endothelial layer of the seed coat, where it would appear related to strengthening seed coat-imposed dormancy and longevity, in addition to protection against pathogens and predators (Debeaujon et al., 2000).

Prodelphinidins (derived from gallocatechin and epigallocatechin units) and procyanidins (derived from catechin and epicatechin units) are predominant types of CTs in legumes (Foo et al., 1996, 1997; Aoki et al., 2000). However, there are significant differences amongst species and genera. Species belonging to *Medicago* and *Trifolium* genera accumulate CTs mainly in flowers and seed coats (Goplen et al., 1980; Pang et al., 2007). On the contrary, *Lotus* spp. show CT accumulation in shoots, roots, flowers and seeds. *Lotus* spp. can differ in quantity and composition of the CTs accumulated, especially in the prodelphinidin/procyanidin/others ratio (Pd/Pc/others).

Shoots and roots from species like *L. pedunculatus, L. uliginosus, L. subbiflorus* and *L. angustissimus* have higher levels of CTs, shoots from *L. corniculatus* have intermediate levels, while roots of *L. corniculatus* and shoots and roots of *L. japonicus, L. filicaulis, L. burtii, L. tenuis, L. krylovii* and *L. schoelleri* have lower CT contents (Sivakumaran et al., 2006; Gruber et al., 2008). It has been also observed that species from the *L. uliginosus* group has a 2–3 fold higher content of Pd than Pc, whereas *L. japonicus* and close relatives contain mainly Pc (Pankhurst and Jones, 1979). Pd has more reactivity than Pc and can exert different biochemical effects, for instance during interaction with proteins (Aerts et al., 1999; Ropiak et al., 2017).

Some authors suggested that the level and/or composition of CTs in roots, along with differential sensitivity of rhizobial strains to CTs, could determine the symbiotic compatibility between *Lotus* spp. and rhizobia (Pankhurst, 1970; Pankhurst and Jones, 1979; Pankhurst et al., 1979, 1982, 1987). Moreover, Cooper and Rao (1992) found that monomeric flavolans, but especially CTs accumulated in Fix^-^ nodules of *L. uliginosus*, in contrast to Fix^+^ nodules. However, these authors also reported that some rhizobial strains were highly effective on their *Lotus* host but exhibited sensitivity to root flavolans. We have also tested the sensitivity of various *Lotus* meso- and bradyrhizobia to *L. uliginosus* CTs, and found no correlation between bacterial sensitivity to these compounds and host-bacteria compatibility (M. J. Lorite, unpublished results).

Transgenic plants of *L. corniculatus* cv. Leo that overexpress *Sn*, a maize *bHLH* transcriptional regulator of anthocyanin biosinthesis, are currently available (Robbins et al., 2003). These plants accumulate different levels of CTs in shoots and roots, and constitute an excellent system to study the effects of these secondary metabolites in rhizobia-host legume specificity. Preliminary works have been carried out to evaluate the CT levels in roots of four *Sn* transgenic plants and its relationship with the symbiosis establishment with *M. loti* NZP2213 and *Bradyrhizobium* spp. NZP2309, in comparison to *L. tenuis* and *L. uliginosus*. Results showed that CT levels were markedly higher in the roots of some of the transgenic plants than in *L. uliginosus*. Nonetheless, all *Sn* transgenic and *L. tenuis* formed Fix^+^ nodules with *M. loti* NZP2213 and Fix^-^ nodules with *Bradyrhizobium* spp. NZP2309 strain, thereby providing evidence that root CT levels do not seem to affect the plant-bacteria compatibility (F. Escaray, unpublished results).

#### Cyanogenic Hydroxynitrile Glucosides

The aminoacid-derived hydroxynitrile glucosides are a large class of bioactive metabolites found in many plant species, some of which may have a role in plant defense against herbivores (Bjarnholt and MØller, 2008). In the legume model *L. japonicus*, four different hydroxynitrile glucosides have been reported: lotoaustralin, linamarin, and the non-cyanogenic rhodiocyanosides A and D (Forslund et al., 2004). At least the first two compounds have been also detected in *L. corniculatus* and other closely related species (Lai et al., 2014). However, production of these compounds has not been observed in *L. uliginosus* and related species (Lai et al., 2014). Although there is no data on a possible relationship of cyanogenic glucosides and symbiosis establishment, it might be worth exploring the coincidental correlation of cyanogenic and non-cyanogenic species with symbiotic incompatibility groups I and II, respectively.

## *Lotus* Rhizobial Inoculants

In **Table 1** we have listed rhizobial strains which are used or recommended for *Lotus* inoculants in various countries. Despite the great diversity of rhizobia establishing effective symbioses with *Lotus* spp., only a reduced number of strains have been used for inoculants worldwide. This is especially true for inoculants involving bradyrhizobia, as one single strain NZP2039 is commonly used as inoculant for *L. pedunculatus* and *L. subibiflorus* in most countries listed. This is partly due to the fact that only few *Lotus* spp. are broadly grown for forage (mainly *L. corniculatus, L. uliginosus, L. tenuis*), and that bacterial strains selected in one country moved to other countries where they were rapidly tested and adopted. Very often too, rhizobia recommended as inoculants for a particular *Lotus* host, were not originally isolated from that host (i.e., NZP2037 isolated from *L. divaricatus*), and perhaps not even from a *Lotus* spp. host (i.e., SU343 could originally have been isolated from a *Lupinus* sp., see below). In some cases, the uncertainty about the bacterial origin can lead to uncertainty about its symbiotic properties. For instance, it is unclear if SU343 (equivalent names NZP 2196, ICMP 3663, ICMP 10808, PDDCC 3663, ATCC 35173, DSM 6046) was originally isolated from *L. corniculatus* in United States (as described for ICMP10808 and mentioned in Howieson et al., 2011), or from *Lupinus* sp. in Australia (as for ICMP 3663/NZP2196 and ATCC 35173, and in Charlton et al., 1981). Therefore it could be considered of unknown origin (as in DSM 6046). This uncertainty can sometimes lead to mistakes and therefore to misuses of a given strain. For instance, Binde et al. (2009) listed strain SEMIA 848 as an inoculant for *L. subbiflorus* in Brazil, however that strain is equivalent to U-510 in Uruguay where it is recommended for *L. corniculatus* and *L. tenuis*. The use of relatively few strains, which often were isolated long ago and brought from distant places, accentuate the need of studies toward selection of efficient rhizobial strains which perform optimally with local cultivars and prevailing environmental conditions. In an interesting study aimed to identify efficient strains for more contemporary forage species such as *L. ornithopodiodes*, Howieson et al. (2011) observed the difficulties to find strains that could optimally perform with all *Lotus* spp. of agronomic interest in New Zealand, because of the large host-strain interaction for symbiotic nitrogen fixation. This poses potential agronomic problems due to conflicts between such specific symbioses. For instance, sowing *L. uliginosus* or *L. subbiflorus* in a field with previous history of *L. corniculatus* can give rise to pasture failures, due to the *L. corniculatus* rhizobia inducing non-fixing pseudonodules in *L. uliginosus*. This further emphasizes the need to explore the existing diversity to select for rhizobial strains that perform optimally under specific conditions.

**Table 1:** Rhizobial strains used for *Lotus* inoculants in different countries.

*Lotus* spp.	Country	Rhizobial strain^a^	Other strain designations	Strain origin	Reference^b^
*L. corniculatus*		LL32			Sannazzaro et al., 2011;
	Argentina	LL12		Argentina	A. Perticari, p.c.
		LL33			
	Brazil	SEMIA 806		Brazil	Roma-Neto et al., 2010;
		SEMIA 816			A.B. de Oliveira, p.c.
	Uruguay	U510	B816/U226	Australia	Sotelo et al., 2011; MGAP
	Australia	SU343	ICMP3663/NZP2196/	Unknown	Charlton et al., 1981;
			ATCC35173/DSM 6046		Howieson et al., 2011
	New Zealand	SU343	ICMP3663	Unknown	ICMP collection
		ICMP9005	ICMP3153/R7A	Unknown	
		B733		Argentina	Sannazzaro et al., 2011;
*L. tenuis/glaber*	Argentina	LL32		Argentina	
		NZP2213		New Zealand	A. Perticari, p.c.
	Brazil	SEMIA 830		Unknown	Roma-Neto et al., 2010
	Uruguay	U510	B816, U226	Australia	Sotelo et al., 2011; MGAP
*L. uliginosus/pedunculatus*	Argentina	U1401	NZP2309	Australia	Lowther, 1984
	Brazil	SEMIA839	NZP2021f/CC829/TAL925	United States	Howieson et al., 2011
	Uruguay	U1401	NZP2309/U526	Australia	Lowther, 1984
	United States	USDA3469	NZP2309	Australia	Barker et al., 2012
	Australia	CC829	NZP2021f	United States	Howieson et al., 2011
	New Zealand	ICMP 5798	NZP2309/CC814s/ICMP4681	Australia	Lowther, 1984;
		ICMP5942	NZP2021f/CC829	United States	Howieson et al., 2011
*L. subbiflorus/hispidus*	Argentina	LL53	CC814b	Australia	A. Perticari, p.c.
	Brazil	SEMIA848	U510	Australia	Binde et al., 2009;
		SEMIA849	U512/NZP2037		A.B. de Oliveira, p.c.
	Uruguay	U531	NC3	Uruguay	MGAP
	Australia	CC814b		Australia	Bullard et al., 2005

*^*a*^It is likely that CC814, CC814s and CC814b correspond to the same original strain. ^*b*^MGAP, Ministerio de Ganaderia, Agricultura y Pesca, Uruguay; ICMP, International Collection of Micro-organisms from Plants, New Zealand*.

The ecological and agronomic potential of the genus *Lotus* for sustainable livestock production and other uses like land reclamation (Escaray et al., 2012), cannot be fully harnessed without acknowledging the great diversity of *Lotus*-rhizobia interactions as well as the specific plant and bacterial requirements, to achieve optimal symbiotic establishment and nitrogen fixation under the increasingly constrained environmental conditions faced by agricultural systems.

## Author Contributions

JS thought out the article, supervised and corrected the writings by all other authors and coordinated discussions. ML and ML-B prepared most work related to taxonomy and diversity, host–bacteria incompatibilities, and constructed the phylogenetic trees, with the support of JM and IVeC, who also collaborated in the section about inoculants. ME (CIC) and AS contributed to the chapter on bacterial compounds involved in specificity and the horizontal gene transfer, whereas FE contributed to the section on plant genetics and host compounds.

## Conflict of Interest Statement

The authors declare that the research was conducted in the absence of any commercial or financial relationships that could be construed as a potential conflict of interest.
