# Supplementary material for: The Rhizobia-Lotus Symbioses: Deeply Specific and Widely Diverse
Source: Front Microbiol. 2018 Sep 12;9:2055. doi: 10.3389/fmicb.2018.02055 (PMC6144797; doi:10.3389/fmicb.2018.02055)
Supplement: Supplementary file 2 [file Image_1.pdf]

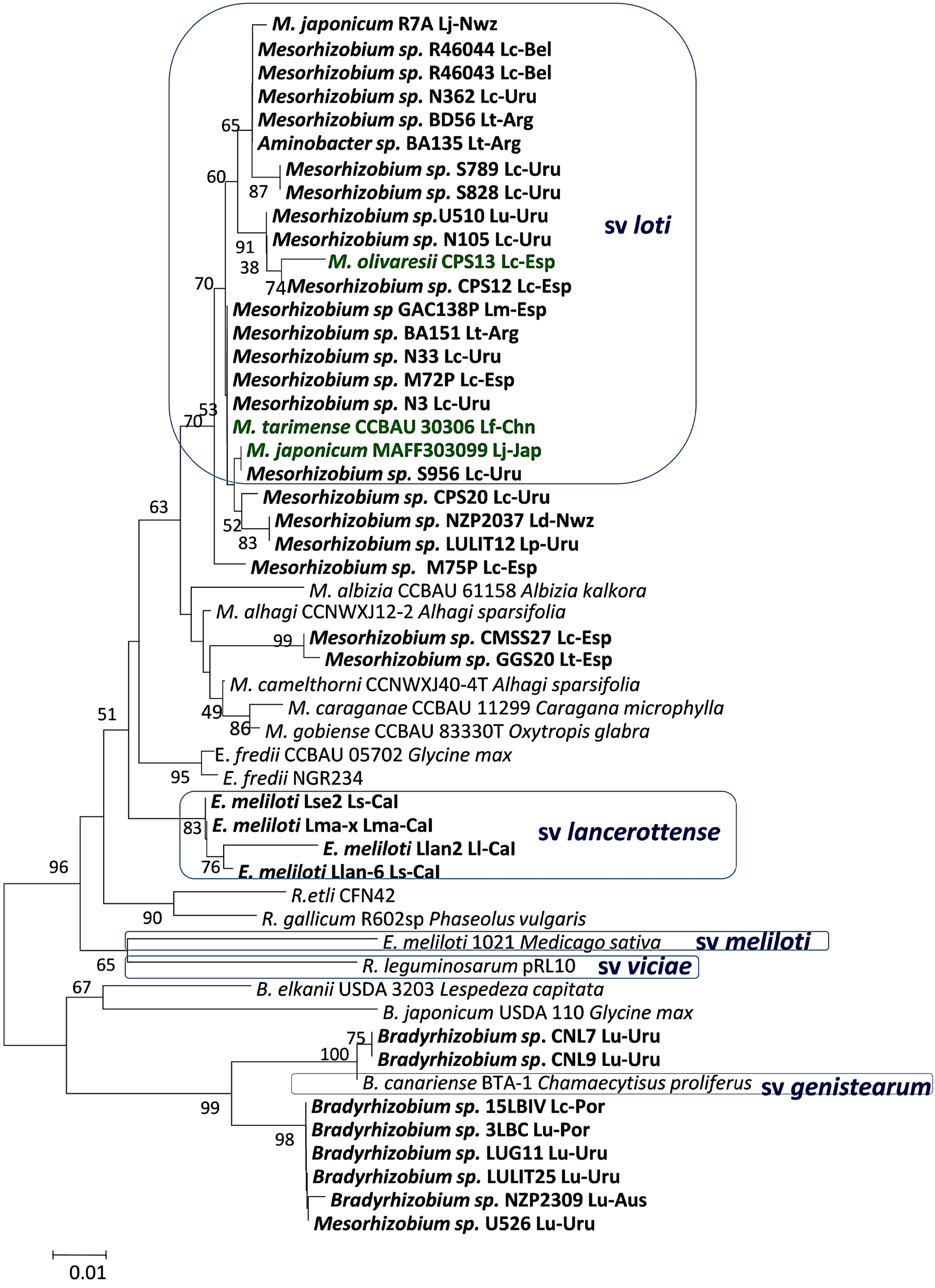

M. japonicum R7A Lj-Nwz
Mesorhizobium sp. R46044 Lc-Bel
Mesorhizobium sp. R46043 Lc-Bel
Mesorhizobium sp. N362 Lc-Uru
Mesorhizobium sp. BD56 Lt-Arg
Aminobacter sp. BA135 Lt-Arg
Mesorhizobium sp. S789 Lc-Uru
Mesorhizobium sp. S828 Lc-Uru
Mesorhizobium sp.U510 Lu-Uru
Mesorhizobium sp. N105 Lc-Uru
M. olivaresii CPS13 Lc-Esp
Mesorhizobium sp. CPS12 Lc-Esp
Mesorhizobium sp GAC138P Lm-Esp
Mesorhizobium sp. BA151 Lt-Arg
Mesorhizobium sp. N33 Lc-Uru
Mesorhizobium sp. M72P Lc-Esp
Mesorhizobium sp. N3 Lc-Uru
M. tarimense CCBAU 30306 Lf-Chn
M. japonicum MAFF303099 Lj-Jap
Mesorhizobium sp. S956 Lc-Uru
sv loti
Mesorhizobium sp. CPS20 Lc-Uru
Mesorhizobium sp. NZP2037 Ld-Nwz
Mesorhizobium sp. LULIT12 Lp-Uru
Mesorhizobium sp. M75P Lc-Esp
M. albizia CCBAU 61158 Albizia kalkora
M. alhagi CCNWXJ12-2 Alhagi sparsifolia
Mesorhizobium sp. CMSS27 Lc-Esp
Mesorhizobium sp. GGS20 Lt-Esp
M. camelthorni CCNWXJ40-4T Alhagi sparsifolia
M. caraganae CCBAU 11299 Caragana microphylla
M. gobiense CCBAU 83330T Oxytropis glabra
E. fredii CCBAU 05702 Glycine max
E. fredii NGR234
E. meliloti Lse2 Ls-Cal
E. meliloti Lma-x Lma-Cal
E. meliloti Llan2 Ll-Cal
E. meliloti Llan-6 Ls-Cal
sv lancerottense
R.etli CFN42
R. gallicum R602sp Phaseolus vulgaris
E. meliloti 1021 Medicago sativa
sv meliloti
R. leguminosarum pRL10
sv viciae
B. elkanii USDA 3203 Lespedeza capitata
B. japonicum USDA 110 Glycine max
Bradyrhizobium sp. CNL7 Lu-Uru
Bradyrhizobium sp. CNL9 Lu-Uru
B. canariense BTA-1 Chamaecytisus proliferus
sv genistearum
Bradyrhizobium sp. 15LBIV Lc-Por
Bradyrhizobium sp. 3LBC Lu-Por
Bradyrhizobium sp. LUG11 Lu-Uru
Bradyrhizobium sp. LULIT25 Lu-Uru
Bradyrhizobium sp. NZP2309 Lu-Aus
Mesorhizobium sp. U526 Lu-Uru
0.01

Figure S1. Phylogenetic tree of *nifH* gene sequences. Bold names correspond to *Lotus* rhizobia, with original *Lotus* species and country of isolation indicated. In bold green are rhizobial species which have *Lotus* symbionts as type strains. Previously defined symbiovars are indicated. Strains with unconfirmed symbiotic phenotypes in *Lotus* are marked with an arrow. Analyses were conducted with MEGA using Neighbor Joining Maximum Likelihood (NJML), for a total of 201 positions. Abbreviations are as in Figure 1.
